# Residence in an Area with Environmental Exposure to Heavy Metals and Neurobehavioral Performance in Children 9–11 Years Old: An Explorative Study

**DOI:** 10.3390/ijerph19084732

**Published:** 2022-04-14

**Authors:** Rocío Capelo, Diane S. Rohlman, Rocío Jara, Tamara García, Jesús Viñas, José A. Lorca, Manuel Contreras Llanes, Juan Alguacil

**Affiliations:** 1 Centro de Investigación en Recursos Naturales, Salud y Medio Ambiente (RENSMA), Universidad de Huelva, 21071 Huelva, Spain; rocio.capelo@dbasp.uhu.es (R.C.); rocio.jara@dqcm.uhu.es (R.J.); tamara.garcia@dqcm.uhu.es (T.G.); manuelj.vinas@juntadeandalucia.es (J.V.); 2 Departamento de Sociología, Trabajo Social y Salud Pública, Universidad de Huelva, 21071 Huelva, Spain; 3 Center for Research on Occupational and Environmental Toxicology, Oregon Health & Science University, Portland, OR 97239, USA; diane-rohlman@uiowa.edu; 4 Departamento de Psicología Clínica, Experimental y Social, Universidad de Huelva, 21071 Huelva, Spain; andres.lorca@dpsi.uhu.es; 5 Consorcio de Investigación Biomédica en Red de Epidemiología y Salud Pública (CIBERESP), 28029 Madrid, Spain

**Keywords:** neurobehavioral test, developmental delay, children, environmental pollution, Spain

## Abstract

We explored the association between residence in an area polluted with metals and neurobehavioral performance in children aged 9 to 11. A cross-sectional study was conducted with thirty boys and thirty girls aged 9 to 11 from public schools in a heavily industrialized area, matched by age (±4 months) and gender with 15 boys and 15 girls from public schools in cities without relevant industrial activity. Neurobehavioral performance was assessed with the Behavioral Assessment and Research System. Linear regression models were used, adjusting for age, sex, social class and multimedia activities to predict each of the neurobehavioral outcome variables. No differences in neurobehavioral performance were found when all children with residence in areas with environmental exposure to metals were classified as exposed and the children from the other provinces as unexposed. However, when we compared children living <1 km from an industrial area with respect to those living more than 1 km away, significant differences were found. Children living <1 km away had lower scores on Finger Tapping (*p* = 0.03), Symbol-Digit (*p* = 0.07) and Continuous Performance (*p* = 0.02) than those living farther away. Our results support the hypothesis that residing close to an area with industrial activity (<1 km) is associated with deficits in neurobehavioral performance among children aged 9 to 11.

## 1. Introduction

Neurodevelopmental disorders, including dyslexia, mental retardation, attention-deficit/hyperactivity disorder, cerebral palsy, autism, and subclinical decrements in brain function, increased significantly in the last ten years and affects around 17% (∼1 out of every 6) of all babies born worldwide with one or more developmental disabilities [1]. Although their etiology is mostly unknown, a small number of industrial chemicals—lead, methylmercury, polychlorinated biphenyls, arsenic, certain pesticides and toluene—are generally recognized as possible causes of such disorders [2,3,4,5,6]. Research on these substances has demonstrated that exposures during early development may cause brain injury at dose levels much lower than those affecting adult brain functions [5,6,7]. In addition to lead and mercury, there is evidence that relates arsenic and manganese exposure to neurodevelopmental problems in children, but there is little information on other contaminants, such as cadmium exposure [8]. The developing nervous system is more susceptible than the adult brain to the disrupting effects of toxic chemicals [5,7]. Infants’ and children’s susceptibility to industrial chemicals is further augmented by increased exposures, higher absorption rates, and the diminished ability to detoxify many exogenous compounds relative to that of adults [5,7,9]. Neurodevelopmental impairment that may result as a consequence of exposure to toxicants represents a continuum, from mental retardation and other clinical syndromes at one extreme to small subclinical deficits in sensory, motor and cognitive impairment at the other [10,11,12,13]. Overt toxicity is usually the consequence of high-level exposure [3,6,11]. Smaller effects fall outside the definition of clinical abnormality and, although less severe at the individual level, may be extremely important to society if large numbers of individuals are exposed [14,15].

Generally, early-life exposure to toxic elements (e.g., As, Cr, Pb, Hg, Cd, U) through water, food and the environment pose major health threats. In this regard, these metals may frequently react with biological systems by losing one or more electrons and forming metal cations, which have an affinity to the nucleophilic sites of vital macromolecules [16,17]. The bioaccumulation of toxic metals leads to a diversity of toxic effects on a variety of body tissues and organs, such as nervous system disorders, gastrointestinal and kidney dysfunction, vascular damage, skin lesions, cancer and immune system dysfunction. High-dose heavy metals exposure, particularly Pb and Hg, may induce severe complications, e.g., kidney failure, bloody diarrhea and abdominal colic pain [18,19]. Chronic exposure may induce human carcinogens [20], while low-dose exposure leads to severe neuropsychiatric disorders, such as anxiety, fatigue and detrimental impacts on intellectual function and intelligence quotient (IQ) in children [21]. Since the 1960s, several industrial estates have been established in the proximity of the Ría of Huelva estuary in SW Spain, which are concentrated in three major industrial parks for chemical and metal industry activities. The main activities include polymetallic sulfide transformation derived from the Iberian Pyritic Belt, petroleum refining, copper smelter, paper manufacture and the production of phosphoric acid from fluorapatite. Specifically, the production of phosphoric acid (an intermediate in the phosphates fertilizer industry) has generated a waste rich in heavy metals and radionuclides called phosphogypsum [22,23]. From 1965 to 2010, the phosphoric acid factories produced approximately 2.5·Mt of PG annually, which has been disposed of in aboveground stockpiles on the salt marshes of the right bank of the Tinto River, less than 1 km from the Huelva city center [22,23]. This dump (1200 ha of surface area and 5 m average height) contains approximately 120 Mt of phosphogypsum, which has caused severe environmental and ecological deterioration in the zone [24,25,26]. These industrial activities, together with the mining activities upstream of the Ria de Huelva estuary, generate huge amounts of waste pollutants, particularly heavy metals, including copper, zinc, cadmium, lead and arsenic, which are delivered to both the fluvial-estuarine system and the air, making this area famous for being one of the most contaminated estuaries in the world in terms of heavy metals [25,26,27,28]. A number of studies have been performed in order to characterize the quality of the air in the city of Huelva [29,30], which have demonstrated a high industrial contribution of SO_2_ with respect to the typical urban emissions, whereas traffic emissions contributed to NO_2_ levels. Arsenic was found to be the main geochemical anomaly in PM_10_. Regarding soil contamination in the fringe of the city of Huelva [31,32], concluding that some areas are potentially contaminated with As, Cd, Cu, Pb, Sb and Zn, exceeding the regulatory levels should be classified as polluted. While a high concentration of As, Cd, Pb and Ni was found in estuarine water surrounding the city of Huelva [23,24,25,26,33]. Biomonitoring studies for metal levels in the Huelva population provide little useful information on whether the pollution is arriving at the general population, as the studies available to date are based on spot urine samples [34,35], which basically reflect the recent intake of food rich in arsenic and other metals, and cannot capture the chronic environmental exposure to most metals.

Neurobehavioral screening test batteries can help identify the early stages of subclinical behavioral or neurological changes and provide a chance to identify major disease processes and persons affected by exposures at stages that offer more effective opportunities for intervention [36,37]. To efficiently assess neurobehavioral performance, children must be old enough to respond to standardized and validated assays. The Behavioral Assessment and Research System (BARS) has been used in environmental exposure studies in several countries and across races/ethnicities, demonstrating cross-cultural reliability and adaptability [38,39]. Recently, the neurobehavioral effects in children have received a considerable amount of attention. The causality of the association between air pollution exposure and negative neurobehavioral outcomes during childhood has been supported by experimental studies, although only some of them used environmentally relevant exposure levels [40]. Some studies reported a deleterious effect of exposures related to living near coal ash storage sites in the USA [41] and others related to living near coal-fired power plants in Canada [42] on different aspects of children’s behavior. Other studies suggested that children living near heavy traffic in Ecuador [39] or areas polluted by mining, metal scrapping and smelters, and e-waste recycling in low and middle-income countries [43] appear to experience subtle neurobehavioral deficits. Even neurobehavioral domains (such as sustained attention, short-term memory and manual-motor speed) and low-level metal exposure in adolescents were observed in recent studies conducted in Belgium [44].

The aim of this study is to evaluate the association between residing in an area that is heavily industrialized and contaminated by mining activities and neurobehavioral performance in children aged 9 to 11.

## 2. Materials and Methods

### 2.1. Participants

A cross-sectional epidemiological study was conducted, and a total of 126 children aged 9 to 11 were recruited from public schools in cities in the province of Huelva (a heavily industrialized area) (*n* = 74; 58.7%) and two populations residing in areas without industrial activity in other Andalusian provinces (Jabalquinto in Jaen and Castilleja de la Cuesta in Seville). All the children recruited in the study were Spanish nationals who were 9 to 11 years old at the time of the test and studying in the 4th to 6th grades at public primary schools. The children had resided in the area of study for at least 5 years and were free from mental retardation and autism spectrum disorder. Of the 126 children, 15 (11.9%) were excluded for lacking biological specimens (urine) for future studies. We matched 60 children (30 boys and 30 girls) from the heavy industrial activity province (Huelva) with 30 children (15 boys and 15 girls) from public schools in the cities without relevant industrial activity by age (±4 months) and gender. The other 21 (16.7%) children were excluded because they failed to complete the BARS test (*n* = 4) or they did not have a pair for the sex and age matching criteria (8 from the Huelva province and 9 from the other provinces). Hence, a total of 90 children were included in the present study.

We recruited children from the following populations in the Huelva province due to their proximity to areas with heavy metal and petrochemical industries (the city of Huelva and Moguer) or their proximity to heavy metal mining (Aracena highlands). Children from cities without known exposure to heavy metals were recruited from an urban residential area near the city of Seville and a rural area where the most important economic activity was related to olive oil production. The social-demographic characteristics for the group of children living in areas with metal pollution and the group of children living in areas without known exposure to heavy metals are presented in Table 1. The response rate from schools in the exposed zones ranged from 12% to 25% (average of 17%), while the unexposed areas schools’ response rate ranged from 35% to 45% (average of 40%).

Based on one question in the questionnaire, we also classified subjects with respect to their exposure status (independently on whether the children’s residence was in an area with known or without known exposure to heavy metals) by asking the parents whether the family residence was located within 1 km of an industrial zone. We also tested for differences in neurobehavioral performance using this strategy to classify children with respect to their exposure status. The socio-demographics for the group of children living in areas located within 1 km of an industrial zone compared to the group of children living in areas more than 1 km of an industrial zone are presented in Table 2.

### 2.2. Exposure Assessment by Urinary Metals Measurement

Urine levels of metals suspected to be related to neurobehavioral toxicity (arsenic, cadmium, lead and mercury) were measured using inductively coupled plasma mass spectrometry (ICP-MS). The detection limits were 0.0399, 0.087, 0.006 and 0.207 µg/g for arsenic, cadmium, lead and mercury, respectively. Results of the comparison of urine metal levels by the study group are shown in Table 3. The eight children with valid urine measurements whose residence was within 1 km of an industrial zone had a median urinary mercury levels over three times higher than children living in areas more than 1 km from an industrial zone, though the difference did not reach statistical significance. However, lead urinary levels were higher among those children living further away (*p* = 0.03; Table 3). A similar pattern of higher urinary mercury was observed when comparing children living in the Huelva area (suspected of having metal pollution) with respect to children from areas without a known metal pollution problem, who showed higher levels of total arsenic but not from inorganic origin (Table 3).

### 2.3. Procedures

We first contacted the director of each of the targeted schools, and after they expressed their willingness to participate, we distributed the study material, which included a letter explaining the study, an informed consent form, and a brief questionnaire to be completed by the children’s parents/guardians that collected parental lifestyle information (social-demographics, smoking and occupation) and information related to the children (medical history, neurobehavioral symptoms, consumption of fish, shellfish and vitamin supplements and physical activity). A teacher from the school distributed the information material among children who met their inclusion criteria. Once the signed informed consent forms were obtained, sessions were organized in the different schools in which a spot urine sample was collected, anthropometric measures (weight and height) were taken, and neurotoxicity was assessed by tests from the Behavioral Assessment and Research System (BARS). The parent’s employment was coded using the National Classification of Occupations 1994 (CNO-1994) and was used as an indicator of the socioeconomic status (SES). The categories for SES included: High SES (I: Professionals with university degree and managers of companies with more than 10 employees; II: Professionals without university degree and managers of companies with less than 10 employees), Medium SES (III: Administrative workers, Self-employed workers (including agriculture); and IVa: Manual skilled workers), and Low SES (IVb: Semiskilled manual workers; and V: Unskilled workers). The parents of all children signed the informed consent. The study protocol was approved by the ethical committee of the Huelva University and was conducted following the Declaration of Helsinki principles.

### 2.4. Neurobehavioral Battery

Neurobehavioral performance was assessed using the Behavioral Assessment and Research System (BARS). This is a computer-based test battery that has the advantage of presenting information in a consistent and efficient manner to all participants while minimizing the impact of the examiner [45]. The BARS battery was developed for use with a broad range of working populations with a variety of education levels and cultural backgrounds [38,39]. The reliability and validity of the tests have been described [46]. Use of the battery with children and specific descriptions of the tests have been previously discussed [47,48]. Briefly, the features of the BARS that enable this broad application include: simple language instructions broken down into basic concepts (step-by-step training with competency testing at each step of instruction); a ‘smiling face’ used to reinforce performance; and adjustable parameter settings [47]. A durable response unit with nine response buttons was placed over a keyboard [45,48] to minimize the impact of working on a potentially intimidating device, such as a computer keyboard. The typical BARS training parameters were applied to ensure that each participant had understood the instructions and was performing adequately before testing began.

The neurobehavioral battery consisted of six computerized tests from the Spanish version of the BARS, including measures of psychomotor functioning (‘Finger Tapping’ and ‘Simple Reaction Time’) and measures of cognitive functioning (‘Symbol-Digit’, ‘Digit Span’, ‘Serial Digit Learning’ and ‘Continuous Performance’). The neurobehavioral test, outcome measures and functions assessed are shown in Table 4. Several tests were composed of separate subtests, with each subtest providing a separate measure. The Tapping Test instructs the participant to press a button as many times as they can for 20 s. The Tapping Test has three subtests—tapping with the preferred hand, tapping with the non-preferred hand, and tapping with Alternate Hands. The number of taps is recorded for all trials. The Symbol-Digit Test presents a matrix at the top of the screen that pairs nine unique symbols with the numbers one through nine. A second matrix that contains only the symbols is shown below. Participants are asked to press the corresponding number button for each symbol. Latencies for each button press are recorded. In the Simple Reaction Time Test, participants are asked to press a button as fast as they can when a square appears on the screen. Participants completed 50 trials, and the latencies for each button press were recorded. The Digit Span Test sequentially presents a series of numbers on the screen in the same sequence (forward) or, in the second part of the test, in the reverse sequence (reverse). Number sequences are presented in increasing lengths starting from three numbers. The Serial Digit Learning Task sequentially presents a nine-digit number on the screen, and the participant is instructed to reproduce the sequence by pressing the nine numbered buttons in the same order. The test terminated after 12 trials or when the participants correctly reproduced the sequence twice in a row. The Continuous Performance Test measures attention. The A-X version of the task was used, where stimuli are sequentially presented on the screen every 50 ms. When the target stimulus (a plus sign followed by a circle) was presented, the participant responded by pressing a button. Three hundred stimuli were presented; 20% of them were target stimuli. The percentages of correct and incorrect and response latencies were recorded. The Continuous Performance Test includes five subtests—Percent of Hits (correctly pressing the button when the target is present), Percent of Correct Rejections (correctly not pressing the button when no target is present), Hit Latency (response latency for total hits), False Alarm Latency (response latency for key presses when no target is present) and D-prime (measure of attentiveness, how well participant discriminates between targets and non-targets). With an examiner present to answer questions, the neurobehavioral tests were administered individually to each child. The testing took place in a regular classroom in each school and generally required approximately 30 min for completion. All tests were administered with instructions in Spanish.

### 2.5. Statistical Analyses

Neurobehavioral performance measures and demographic variables, such as age, weight and height, were summarized using means and standard deviations. The tests chosen have been used in prior studies and include tasks that would be affected by the functional deficits seen in children with early-life exposure to neurotoxicants [38,39,49]. Linear regression models were used for each one of the fourteen separate dependent variables (see Table 5, Table 6, Table 7 and Table 8), representing independent aspects of performance on the BARS battery. Multivariate models included covariates, which were reported to be important in the neurobehavioral development of children (age (quantitative continuous), gender, socioeconomic status, number of hours a day spent on multimedia activities) and the exposure variable to predict each of the neurobehavioral outcome variables. Further adjustment for ‘smoking before their children’, weight, height or BMI did not change the risk estimates of the models. A *p*-value <0.05 was considered as statistically significant.

## 3. Results

### 3.1. Comparison of Scores on Neurobehavioral Toxicity between the Group of Children with Residence in Areas with Environmental Exposure to Heavy Metals and the Group of Children Living in Areas without Known Presence of Heavy Metals

#### 3.1.1. Psychomotor Tests

The performances on the majority of psychomotor test outcomes (all the tapping measures) were similar between children with residence in areas with environmental exposure to heavy metals and children with residence in areas without the known presence of heavy metals. Although not significant, children with residence in the exposed areas performed slightly worse on the two simple reaction time measures (latency = 426.98 and 408.46 ms (*p* = 0.12); total errors = 1.97 and 1.50 (*p* = 0.53)) than children with residence in the areas without known presence of heavy metals (Table 5).

#### 3.1.2. Cognitive Tests

The performances on the cognitive tests (‘Symbol Digit’, ‘Serial Digit Learning’ and ‘Digit Span’ and most of the ‘Continuous Performance Test’ measures) were roughly similar between the two groups, although children of the exposed group had faster (better) continuous performance test latencies (hit latency = 347.98 ms) than children of the areas without known presence of heavy metals (381.64 ms; *p* = 0.12) (Table 6).

### 3.2. Comparison of Scores on Neurobehavioral Toxicity between Children Living within 1 km of an Area with Industrial Activity versus Children Living More Than 1 km from an Area with Industrial Activity

#### 3.2.1. Psychomotor Tests

Performance on the ‘tapping with preferred hand’ measure of the ‘Finger Tapping Test’ revealed differences between the two groups. Children living more than 1 km away from an area with industrial activity perform better than children living within 1 km of an area with industrial activity (number of taps = 75.57 and 68.17 (*p* = 0.03), for children living more than 1 km from an area with industrial activity and children living further away, respectively). The differences in the other tapping measures were not statistically significant, although the group of children living more than 1 km performed better on the two other remaining finger tapping measures (‘tapping with non-preferred hand’, number of taps = 64.21 and 57.27 (*p* = 0.09); ‘tap alternating hands’, number of taps = 38.05 and 33.80 (*p* = 0.35), for children living more than 1 km away from an area with industrial activity and children living within 1 km of an area with industrial activity, respectively). The performance on the other psychomotor test (‘Simple Reaction Time’) was similar between the two groups (Table 7).

#### 3.2.2. Cognitive Tests

Only the ‘continuous performance hit latency’ test showed significant differences between the two groups. Children living more than 1 km away from an area with industrial activity scored faster (better) on continuous performance test latencies’ hits (hit latency = 348.35 ms) than children living within 1 km (hit latency = 418.96 ms, *p* = 0.02) (Table 7). The ‘continuous performance false alarm latency’ and the ‘symbol digit latency’ tests did not show statistically significant differences, but children living more than 1 km away also performed better (false alarm latency = 435.83 ms) than children living within 1 km of an area with industrial activity (486.47 ms; *p* = 0.17); symbol digit latency = 3293.31 and 3528.77 ms; *p* = 0.07). The results of the remaining continuous performance test measures and the other cognitive test (‘Serial Digit Learning’ and ‘Digit Span’) were similar between the two groups (Table 8).

## 4. Discussion

We found an association between living within 1 km of an area with industrial activity and both psychomotor and cognitive tests, which evaluate response speed, motor coordination, complex mental function and attention. However, no differences were found in the neurobehavioral development assessment tests when comparing the group of children with residence in an area with environmental exposure to heavy metals with the group of children who lived in areas without environmental pollution of heavy metals. These results are congruent with the view that the variable ‘residency on the proximity to an industrial area’ might be a better choice for epidemiological environmental association studies than using the variable ‘residence on an area with environmental exposure’ [34,36,37]. In fact, human exposure to metals has been reported both in Spain and other countries in children living in the vicinity of smelters and mining areas [35,38,39,40,41,42], and several studies have shown an adverse relationship between heavy metals exposure and neurobehavioral performance. Thus, Lozano et al. [50] reported that Hg exposure is associated with poorer neurobehavioral development, worse processes related to mental agility, sequential organization, short-term memory and visual memory, all of which are related to the attention process, in 9 and 11-year-old Spanish children. Sex and the presence of certain genetic polymorphisms modified this association. Another study with Mexican schoolchildren found an association between arsenic urine levels with several cognition tests, which represent complex cognitive processes, such as memory, problem solving and attention [51]. Arsenic is a known neurotoxicant that affects the peripheral nervous system [52,53] and central nervous system [54]. The peripheral neuropathy caused by chronic or subacute arsenic exposure is well documented [53]. However, the effects of arsenic to chronic low-level exposure on the central nervous system are rarely reported. At least one study has shown that exposure to arsenic is associated with deficits in cognitive performance among Cambodian school-age children, even at low exposure levels, affecting complex cognitive processes, such as memory and problem solving [55]. In our study, despite total arsenic being higher among the unexposed subjects, inorganic arsenic levels were similar between exposure groups, which highlights the importance of arsenic speciation when analyzing arsenic in urine samples. Our urine metal analysis suggests that the most likely suspected metal responsible for the association found could be mercury rather than arsenic.

The differences found in the Finger Tapping Test are compatible with impairment in the control and coordination of distal muscle groups in the upper limbs. The movement involved in single-finger tapping is complex and can be affected by perception senses and emotional and physical health. A previous study using the BARS system also showed differences in finger tapping performance in children exposed to pesticides known to be neurotoxicants [49]. The differences found for the continuous performance test indicate impairment of sustained attention, the ability to maintain a consistent focus on some continuous activity or stimuli, and is associated with impulsivity. A recent study among children 6–14-years-old living near an industrial coal-fired power plant found an association between PM_10_ and continuous performance test, but not for the latency time task, as we did [42]. A cross-sectional study that included some children from our study area found associations between arsenic [56], cadmium [57] and chromium [58] with different neurobehavioral endpoints. An American matched case-control study found differences in the scores of a neuropsychological test between children with low and high levels of manganese exposure. Deficits mainly occurred in the domains of attention and motor control, possibly due to neurotoxicity involving basal ganglia and forebrain regions [59]. Extensive research has identified specific lead-associated neurobehavioral deficits showing consistent neurobehavioral deficits in relation to low levels of lead exposure, as reported in another American study [60]. These deficits were found in the domains of intelligence, reaction time and attention. Furthermore, they found that none of these neurobehavioral outcomes showed evidence of a threshold below which blood lead levels appear to be ‘safe’. Lead neurotoxicity is mainly associated with central nervous system dysfunction. It is widely known that high levels of lead exposure can result in adverse neurocognitive and behavioral consequences in children [61,62]. Deficits on tests of intellectual function, increased distractibility, short attention span, hyperactivity and impaired school performance are several examples of types of behavioral deficits already observed in Spanish children after developmental exposure to lead [35]. Lower blood levels of lead are also neurotoxic in children and have lasting effects on neurobehavioral functioning [63]. However, in our study, as urinary lead was higher among those children living away from the industrial zone, it is unlikely to be responsible for the associations found.

The underlying mechanism for metal neurobehavioral toxicity is not well understood. Evidence from numerous sources demonstrates that neural development lasts from the embryonic period through adolescence [64]. While most of the basic structure is laid down before birth, neuron proliferation and migration continue in the postnatal period. The blood–brain barrier is not fully developed until the middle of the first year of life. The number of synaptic connections between neurons reaches a peak around the age of two and then falls by about half. Similarly, there is a great deal of postnatal activity in the development of the receptors and transmitter systems, as well as in the production of myelin [65]. Synaptogenesis and myelination continue through puberty [64]. Many of the toxic agents known to damage the developing brain interfere with the processes involved in its development [65]. Thus, the developing nervous system is more susceptible to the disrupting effects of toxic chemicals than the adult brain is. Levels of exposure that produce few or no obvious effects on the mature nervous system in adults may pose a serious risk to the developing nervous system [7]. Studying children aged between 9 and 11 years old provides an acceptable equilibrium between the likelihood of unveiling subclinical neurological disorders and the children’s capability to perform the neurobehavioral tests.

Our findings must be interpreted in light of several limitations. First, because of the cross-sectional nature of the study, we cannot be certain of the causal direction of the associations observed. However, the large body of research on laboratory animals and prospective human studies demonstrating similar associations renders the inference that the children’s environmental exposure to heavy metals preceded the onset of their neurodevelopmental impairments as plausible. Moreover, the polluted areas had a pollution problem for more than 11 years (older age of any of our participants), and our inclusion criteria included living in the same address for more than 5 years, and we excluded children with neurobehavioral problems, such as mental retardation and autism spectrum disorder. Second, children living in an area with environmental pollution of heavy metals do not necessarily imply that all children living in that area have accumulated an important burden of metals in their bodies. This study was designed to compare children living in an area with environmental pollution of heavy metals (i.e., the province of Huelva) with children from areas without this type of pollution and assumes that the former is a group at risk because of their exposure to heavy metals (an indirect exposure measurement). We relied on a number of studies that have been performed to characterize the quality of the air [29,30], soil [31,32], water [23,24,25,26,33] and sediments [27,28] in the city of Huelva. Relatively higher concentrations of phosphate, arsenic, copper, zinc and lead in total suspended particulate [29] and PM_10_ have been reported in the air of Huelva than in other cities in Spain [29,66]. Oliveira et al. [67] have registered high As^3+^ in atmospheric particulate matter [30] derived from industrial emissions. With respect to the air PM_2.5_ geographical distribution in Huelva, the Cu-smelter factory and the sea breeze circulation are the main factors controlling the impact of the Cu-smelter on the air quality of the city [29]. Such a pattern should have a higher contribution to the metal exposure related to the copper smelter to the children of Huelva with respect to children from outside Huelva. To date, the only study on human data conducted in the study area showed no differences in arsenic urine levels between subjects aged 5 to 17 from Huelva city with respect to the other Andalusian capital cities [34]. Such a finding is not surprising if we take into account that investigators measured total arsenic in urine, which is basically a marker of organic arsenic, instead of inorganic arsenic, which is the one related to toxicity in humans, and released by the industry. Third, even if the exposure occurs, deficits may not show up until adolescence. There is frequently a long latency period between the exposure and when any effects are observable or measurable. For instance, most intellectual deficits are not apparent until the individual encounters academic settings in later childhood or adolescence [14,65]. Fourth, socioeconomic status (SES) has been shown to be a determinant of cognitive ability and achievement from early childhood through young adulthood [68]. SES can also determine whether a family lives in close proximity to industrial areas [69]. However, in our study, SES was higher in the group of children living in the study area with environmental exposure to heavy metals than in the control group (Table 1), and SES was also higher in the group of children living less than 1 km from an area with industrial activity than the group of children living more than 1 km away (Table 2). This difference would either tend to dilute the possible effects of contamination or go in the opposite direction; as concerned, educated parents may observe slightly slower development in their children and participate for that reason. Children with higher experience using multimedia devices can perform better with the BARS test. SES might also be related to the ‘number of hours per day spent on multimedia activities’, which was accounted for in the multivariate analyses. Further, smoking before the children might be related to a lower SES and a higher likelihood of exposure to metals. However, we collected information on the smoking habits of the parents and whether they smoked before their children at home, and the risk estimates did not differ. Fifth, the relatively low and different response rates among the group of children living in the study area with environmental exposure to heavy metals (17%) and the control group (40%) could indicate a potential selection bias if the reason not to participle on the study was related to either exposure or neurobehavioral performance. As in many epidemiological studies, we cannot know the reasons for which subjects decided not to participate; however, as it was not known by the general public that the possibility of an association between metals and neurobehavioral toxicity, and because we adjusted the results by SES, we believe it unlikely to have affected our study results. Sixth, comparisons were conducted on a relatively limited number of children, thus considerably restricting this study’s statistical power. With 60 children from exposed areas and 30 children from unexposed areas, we have 80% power to detect differences between means of the effect parameters of a magnitude of 63% of their standard deviation (SD). Hence, negative results should be interpreted with caution for differences between means lower than 63% of the SD, as we could not be detecting possible real associations. The lack of statistical power cannot explain the possible false positive association identified in our study between residing in close proximity to an area with industrial activity and deficits in the Continuous Performance Test and the Finger Tapping Test. Seventh, we collected urine instead of blood for the biomarker analysis for logistic reasons, as urine is more easily collected, and parents are more likely to allow their children to participate in epidemiological studies. The NHANES Population-based Biomonitoring study reports data on urine levels for arsenic, cadmium, lead and mercury, though for the three latter, they also provide information on blood levels [70]. For lead and mercury, urine levels may reflect more recently absorbed exposure, greater individual variation and greater potential for contamination [70], for which blood levels are usually preferable.

Despite the discussed limitations, our study also includes some strengths. First, this is the first study that analyzes the association between potential exposure to industrial contamination and neurobehavioral effects in the province of Huelva on an individual basis. Second, the neurobehavioral function was assessed with the BARS computerized test battery, which is a validated instrument based on the implementation of tests to provide a series or battery of neurobehavioral tests optimized for the detection of neurotoxicity in human populations. This test was also adapted for use with children [47]. Third, demographic variables, such as age, gender and socioeconomic status, have been known to impact performance on the neurobehavioral test [71]. These variables were controlled for in the analysis. Fourth, the neurobehavioral tests were administered on a computer, which may also affect performance. The computer experience has been shown to impact performance on cognitive tests [46]. We collected information on the number of hours a day spent on multimedia activities completed, which revealed no differences between the groups of children. Fifth, as exposures to environmental neurotoxic agents for different lengths of time could potentially produce differential effects [14], in this study, we restricted children’s inclusion to those who had resided in the area of study for at least 5 years. Thus, we guarantee at least 5 years of environmental exposure time for the study population. Sixth, our findings for the Continuous Performance Test and for the Finger Tapping Test were consistent with the results from the other tests of the same battery, not showing statistically significant differences but suggesting a worse performance in the exposed group.

## 5. Conclusions

Our results support the hypothesis that residing in close proximity (less than 1 km) to an area with industrial activity is associated with deficits in neurobehavioral performance among children aged 9 to 11. Given the cross-sectional nature of the study and the study limitations, other studies are needed to replicate the neurobehavioral deficits seen here before definitive conclusions can be drawn about the effects of pollution with heavy metals in the province of Huelva on neurobehavioral performance among children. Despite these limitations, this study is an important first step in understanding the patterns of child development in this population and raises important questions regarding the impact of the industry on health in this region.

## Figures and Tables

**Table 1:** Social-demographic characteristics of the group of children with residence in areas with environmental exposure to heavy metals and the group of children living in areas without known presence of heavy metals.

	Areas with Metal Pollution				Areas without Known Metal Pollution				
	*n*	%	Mean	SD	*n*	%	Mean	SD	*p* *
Sex									
Boys	30	50.0			15	50.0			1.00
Girls	30	50.0			15	50.0			
Age			10.2	0.62			10.1	0.63	0.51
Antropometric variables									
Weight (kg)			41.4	10.5			38.8	10.1	0.28
Height (m)			1.43	0.07			1.41	0.08	0.12
Place of residence									
Huelva	41	68.4			-	-			
Aracena highlands (Huelva)	8	13.3			-	-			
Moguer (Huelva)	11	18.3			-	-			
Castilleja (Seville)	-	-			15	50.0			
Jabalquinto (Jaen)	-	-			15	50.0			
Socioeconomic status									
High (I, II)	18	30.0			2	6.70			0.04
Medium (III, IVa)	33	55.0			22	73.3			
Low (IVb, V)	9	15.0			6	20.0			

* *p*-value based on the Chi-Square (proportions) or Student’s *T* (means) tests.

**Table 2:** Social-demographic characteristics of children living within 1 km from an area with industrial activity and children living more than 1 km away.

	Residence < 1 km				Residence > 1 km				
	*n*	%	Mean	SD	*n*	%	Mean	SD	*p* *
Sex									
Boys	4	26.7			41	54.7			0.048
Girls	11	73.3			34	45.3			
Total	15	100			75	100			
Age			10.1	0.76			10.2	0.60	0.866
Antropometric variables									
Weight (Kg)			39.4	9.06			40.7	10.7	0.645
Height (m)			1.42	0.07			1.43	0.08	0.816
Place of residence									
Huelva	12	80.0			29	38.7			0.047
Aracena highlands (Huelva)	-	-			8	10.7			
Moguer (Huelva)	1	6.7			10	13.3			
Castilleja (Seville)	-	-			15	20.0			
Jabalquinto (Jaen)	2	13.3			13	17.3			
Socioeconomic status									
High (I, II)	7	46.7			13	17.3			0.018
Medium (III, IVa)	8	53.3			47	62.7			
Low (IVb, V)	0	0.00			15	20.0			

* *p*-value based on the Chi-Square (proportions) or Student’s T (means) tests.

**Table 3:** Urinary metal levels of children by environmental exposure classification variable.

	Residence Distance to Industrial Areas			Areas with Metal Pollution	Areas w/o Known Metal Pollution	
	<1 km *n* = 8	>1 km *n* = 57		*n* = 49	*n* = 20	
Urine Mtal Levels (mg/g)	Median	Median	*p* *	Median	Median	*p* *
Total arsenic	22.7	26.8	0.62	23.8	62.3	0.05
Total arsenic/creatinine	42.7	42.7	0.70	41.4	73.5	0.10
Inorganic arsenic	1.96	1.86	0.80	1.86	2.19	0.35
Inorganic arsenic/creatinine	4.82	3.11	0.21	4.00	3.66	0.82
Total cadmium	0.11	0.14	0.54	0.14	0.18	0.29
Total cadmium/creatinine	0.25	0.22	0.69	0.23	0.25	0.60
Total mercury	9.93	4.22	0.13	6.54	2.64	0.08
Total mercury/creatinine	29.4	6.99	0.11	10.6	4.38	0.04
Total lead	0.61	1.81	0.02	1.43	1.86	0.28
Total lead/creatinine	0.56	3.03	0.03	2.06	3.24	0.57

* Mann–Whitney *U* test.

**Table 4:** Neurobehavioral tests, outcome measures and functions tested in the battery.

Neurobehavioral Test	Outcome Measure	Function
Finger tapping	Number of taps	Response speed, coordination
Symbol-digit	Latency	Coding, complex functioning
Simple reaction time	Latency	Response speed
Digit span	Correct score	Attention, memory
Serial digit learning	Score	Learning
Continuous performance test	Percent hits, percent false alarms, percent omissions, d-prime	Sustained attention

**Table 5:** Comparison of neurobehavioral performance on psychomotor functioning between the group of children with residence in areas with environmental exposure to metals and the group of children with residence in areas without known exposure to metals.

	Areas with Metal Pollution (*n* = 60)		Areas without Known Metal Pollution (*n* = 30)				
	Mean	SD	Mean	SD	β *	*p*	
Finger tapping							
Number of taps							
Tapping with preferred hand	74.2	11.3	74.1	11.3	−0.68	0.77	Better
Tapping with non-preferred hand	61.5	11.2	63.6	10.2	2.30	0.29	Better
Tapping with alternating hands	35.2	11.4	38.3	11.7	2.62	0.30	Better
Simple reaction time							
Latency (ms)	408	51.0	427	61.8	20.3	0.12	Worse
Total errors	1.50	1.88	1.97	3.02	0.40	0.53	Worse

* GLM models adjusted for age, sex, social class and number of daily hours on multimedia activities.

**Table 6:** Comparison of neurobehavioral performance on cognitive functioning between the group of children with residence in areas with environmental exposure to metals and the group of children with residence in areas without known exposure to metals.

	Areas with Metal Pollution (*n* = 60)		Areas without Known Metal Pollution (*n* = 30)				
	Mean	SD	Mean	SD	β *	*p*	
Serial digit learning							
Score	9.55	7.55	10.7	7.04	0.72	0.67	Better
Digit span							
Score							
Forward	4.67	0.96	4.61	0.85	−0.05	0.82	Better
Reverse	3.90	1.00	3.78	0.84	−0.17	0.47	Better
Continuous performance							
Percent of hits	0.79	0.18	0.78	0.16	−0.02	0.68	Better
Percent of correct rejections	0.92	0.06	0.91	0.07	−0.02	0.34	Better
Hit latency (ms)	382	88.5	348	89.0	−30.9	0.12	Worse
False alarm latency (ms)	436	108	446	121	11.6	0.68	Worse
d-Prime	2.56	0.99	2.47	0.95	−0.14	0.52	Better

* GLM models adjusted for age, sex, social class and number of daily hours on multimedia activities.

**Table 7:** Comparison of neurobehavioral performance on psychomotor functioning between children living within 1 Km of an area with industrial activity and children living more than 1 Km away.

	Residence < 1 km (*n* = 15)		Residence > 1 km (*n* = 75)				
	Mean	SD	Mean	SD	β *	*p*	
Finger tapping							
Number of taps							
Tapping with preferred hand	68.2	11.7	75.6	10.8	−6.45	0.03	Better
Tapping with non-preferred hand	57.3	9.12	64.2	10.5	−4.76	0.09	Better
Tapping with alternating hands	33.8	7.80	38.1	12.3	−3.12	0.35	Better
Simple reaction time							
Latency (ms)	436	70.5	418	56.9	10.1	0.55	Worse
Total errors	1.87	2.90	1.85	2.71	−0.18	0.83	Worse

* GLM models adjusted for age, sex, social class and number of daily hours on multimedia activities.

**Table 8:** Comparison of neurobehavioral performance in cognitive functioning between children living within 1 Km of an area with industrial activity and children living more than 1 Km away.

	Residence < 1 km (*n* = 15)		Residence > 1 km (*n* = 75)				
	Mean	SD	Mean	SD	β *	*p*	
Serial digit learning							
Score	11.6	7.08	9.92	7.26	1.17	0.59	Better
Digit span							
Score							
Forward	4.60	0.63	4.58	1.08	0.08	0.80	Better
Reverse	3.27	1.49	3.21	1.66	0.12	0.80	Better
Continuous performance test							
Percent of hits	0.81	0.11	0.77	0.17	0.01	0.79	Better
Percent of correct rejections	0.91	0.06	0.92	0.07	−0.01	0.51	Better
Hit latency (ms)	419	96.4	348	85.8	60.2	0.02	Worse
False alarm latency (ms)	486	136	436	112	51.8	0.17	Worse
d-Prime	2.50	0.90	2.45	0.94	−0.05	0.86	Better

* GLM models adjusted for age, sex, social class and number of daily hours on multimedia activities.

## Data Availability

The data presented in this study are available on request from the corresponding authors. The data are not publicly available due to ethical.
